# ZrgA contributes to zinc acquisition in Vibrio parahaemolyticus

**DOI:** 10.1080/21505594.2022.2156196

**Published:** 2023-01-04

**Authors:** Chengkun Zheng, Jun Qiu, Yimeng Zhai, Man Wei, Xiaohui Zhou, Xinan Jiao

**Affiliations:** a Jiangsu Key Laboratory of Zoonosis, Yangzhou University, Yangzhou, China; b Joint International Research Laboratory of Agriculture and Agri-Product Safety, the Ministry of Education of China, Yangzhou University, Yangzhou, China; c School of Public Health and Emergency Management, Southern University of Science and Technology, Shenzhen, P.R. China

**Keywords:** *Vibrio parahaemolyticus*, zinc acquisition, ZrgA, Zur, motility, virulence

## Abstract

Metals are nutrients essential for almost all lifeforms. Bacteria have evolved several mechanisms to overcome the metal restrictions imposed by the host. *Vibrio parahaemolyticus* causes severe threats to public health and significant economic losses in shrimp aquaculture. Herein, we report that ZrgA contributes to zinc acquisition in this pathogen. The operon *VP_RS01455* to *VP_RS01475* of *V. parahaemolyticus* encodes the putative Zn transporter ZrgABCDE, whose homologs are widely distributed in *Vibrionaceae*. RNA sequencing analysis revealed that *V. parahaemolyticus* modulates the transcriptome in response to Zn limitation. Genes in the Zinc uptake regulator (Zur) regulon are upregulated during Zn limitation, including three genes annotated to encode Zn-binding proteins. Significant upregulation of these three genes during Zn limitation was also confirmed by quantitative real-time PCR (qRT-PCR) analysis. However, only the mutants containing a *VP_RS01470* (*zrgA*) deletion exhibited impaired growth under Zn-deficient conditions, indicating that *VP_RS01470* plays the predominant role in *V. parahaemolyticus* Zn acquisition. The *VP_RS01470* deletion mutant displayed a false appearance of decreased swimming motility under Zn-deficient conditions, as revealed by the fact that the polar flagellar-related genes were not downregulated in the mutant. Moreover, *VP_RS01470* deletion produced no noticeable impact on the swarming motility and virulence in mice. qRT-PCR analysis and β-galactosidase activity assays indicated that Zur negatively regulates *VP_RS01470* expression in *V. parahaemolyticus*. Collectively, our findings suggest that ZrgA is required for Zn acquisition in *V. parahaemolyticus* and highlight the importance of detecting the expression of flagellar genes during analysis of motility of a mutant deficient in growth.

## Introduction

*Vibrio parahaemolyticus* is a ubiquitous Gram-negative bacterium in a wide variety of environments, including oceans, coasts, and estuaries [1]. Acute gastroenteritis can often be attributed to the ingestion of seafood contaminated by this bacterium [2]. In addition, *V. parahaemolyticus* is occasionally associated with several other diseases, such as septicaemia, necrotizing fasciitis, and wound infections [3]. In 1950, a foodborne disease caused by this pathogen was reported in Japan, which resulted in 272 illnesses with 20 deaths [1]. Subsequently, this pathogen has been isolated worldwide in either sporadic cases or outbreaks of gastroenteritis [4]. In China, *V. parahaemolyticus* represents the most prevalent foodborne pathogen in food commodities; while in America, it is the major cause of gastroenteritis associated with seafood [5,6]. Moreover, this bacterium causes acute hepatopancreatic necrosis disease in shrimp and thereby responsible for significant economic losses in shrimp aquaculture [7,8]. *V. parahaemolyticus* utilizes a large number of virulence factors during infection to cause cytotoxicity and enterotoxicity [2]. Despite some progress, extensive research is still required to reveal the physiology and pathogenesis of this bacterium.

Metals are nutrients essential for almost all lifeforms. Many proteins need to interact with metal to maintain structural stability and/or exert their function [9]. To defend against invading pathogens, the mammalian hosts have evolved a strategy termed nutritional immunity, i.e. sequestration and mobilization of metals, which renders them unavailable to the pathogens [10]. For example, calprotectin, a host metal-binding protein released by neutrophils at sites of infection, can sequester manganese (Mn) and zinc (Zn), thus inhibiting bacterial proliferation [11,12]. However, bacterial pathogens have developed several mechanisms as countermeasures to combat the host-imposed nutritional immunity [13]. For instance, *Yersinia pestis* produces yersiniabactin to repress the Zn-mediated nutritional immunity of the mammalian and insect hosts during infection [14]. Despite their essential role, metals are toxic to cells when accumulated in excessive amounts [15]. Moreover, the hosts exploit metal toxicity to control bacterial infection [10]. To respond to restricted or excess metals, bacteria can regulate the expression of specific genes through metalloregulators [13].

Metal acquisition and the impacts of metals on the physiology and pathogenesis of *V. parahaemolyticus* have been partly revealed. A previous study showed that iron (Fe) and Mn enhance *V. parahaemolyticus* virulence in mice [16]. Calcium (Ca) and Fe have been reported to modulate type III secretion and swarming motility in *V. parahaemolyticus* [17]. Nuclear magnetic resonance-based metabolomics revealed that ferric iron (Fe[III]) stimulation in *V. parahaemolyticus* upregulates 21 metabolites and downregulates nine [18]. Interestingly, *V. parahaemolyticus* has adopted strategies to obtain Fe from host sources, including the utilization of siderophores, xenosiderophores, proteases, and iron-protein receptors [19]. Zn ranks second in terms of the content of transition metals in living beings, behind only Fe; approximately 5% to 6% of the total proteins encoded in bacteria are Zn-binding proteins [20]. A horizontally acquired ZnuA homolog, here referred to as ZnuAII, contributes to Zn transport and virulence in *V. parahaemolyticus* [21]. However, little is known about the global transcriptome responses of *V. parahaemolyticus* to Zn limitation. Indeed, it remains unclear if additional Zn transporters exist in this bacterium.

In this study, the adaptive responses of *V. parahaemolyticus* to Zn limitation were explored by RNA sequencing analysis. *VP_RS01470*, a gene negatively regulated by the Zn uptake regulator (Zur), was found to play the predominant role in Zn acquisition in *V. parahaemolyticus*.

## Materials and methods

### Bacterial strains, plasmids, primers, and culture conditions

Bacterial strains and plasmids are listed in Table S1; primers are listed in Table S2. *V. parahaemolyticus* RIMD2210633 [22] was used as the wild type (WT) strain. Unless otherwise specified, all *V. parahaemolyticus* and *Escherichia coli* strains were grown at 37°C in lysogeny broth (LB) with constant shaking at 220 rpm or on LB agar. The Zn chelator N,N,N’,N’-Tetrakis (2-pyridylmethyl) ethylenediamine (TPEN, dissolved in DMSO; Sigma-Aldrich) was used to prepare Zn-deficient medium [23]. When required, either carbenicillin (50 μg/mL), chloramphenicol (25 μg/mL), or isopropyl β-D-1-thiogalactopyranoside (IPTG; 1 mM) was added.

### RNA extraction

The RIMD2210633 strain was grown to an OD_600_ of 0.7 (early-exponential phase). Three aliquots (1 mL) were removed and centrifuged to collect bacterial cells. The three bacterial pellets were resuspended with 1 mL LB pretreated with either DMSO, 35 μM TPEN, or 35 μM TPEN along with 500 μM Zn, respectively. After incubating at 37°C for 15 min, the cultures were centrifuged to collect the cells. Total RNA was extracted using the Eastep Super Total RNA Isolation Kit (Promega). Three biological replicates, obtained from three independent experiments, grown in each of the conditions of interest were used for RNA extraction. After evaluating RNA integrity and RNA concentrations, the RNA samples were used for RNA sequencing and quantitative real-time PCR (qRT-PCR) analysis.

In another experiment, the WT, Δ*1470*, and CΔ*1470* strains were grown to an OD_600_ of 0.7. Then, bacterial cells from 1 mL of each culture were collected and resuspended with 1 mL LB pretreated with 35 μM TPEN. After incubating at 37°C for 15 min, bacterial cells were collected for RNA extraction. Three biological replicates, obtained from three independent experiments, grown in each of the conditions of interest were used.

Additionally, the WT, Δ*zur*, and CΔ*zur* strains were grown to an OD_600_ of 0.7. Subsequently, bacterial cells from 1 mL of each culture were collected for RNA extraction. Three biological replicates, obtained from three independent experiments, grown in each of the conditions of interest were used.

### RNA sequencing analysis

Ribosomal RNA depletion, cDNA library preparation, and sequencing were performed at Sangon Biotech Co. Ltd., as previously described [24]. The clean reads were mapped to *V. parahaemolyticus* RIMD2210633 genome using Bowtie2. Gene expression was estimated by TPM (transcripts per million) using featureCounts. The genes with a fold change greater than 2 and an adjusted *P* value (*q* value) less than 0.05 were defined as the differentially expressed genes (DEGs). KEGG pathway enrichment analysis was performed using KOBAS-i [25]; the pathways with a *q* value less than 0.05 were considered significantly enriched. RNA sequencing data have been submitted to Gene Expression Omnibus (GEO) under accession no. GSE200927.

### Reverse transcription-PCR (RT-PCR) analysis

Total RNA extracted from the RIMD2210633 strain was used to synthesize cDNA as previously described [24]. The resulting cDNA was amplified using primers 1455-F and 1475-R. PCR amplification of the genomic DNA (gDNA) from RIMD2210633 and cDNA- (cDNA reaction without reverse transcriptase) were used as positive and negative controls, respectively.

### qRT-PCR analysis

qRT-PCR analysis was performed as previously described [24]. The PCR efficiency for each primer pair was assessed by qRT-PCR analysis of serially diluted gDNA (1:10 dilution) as previously described [26]. Only the primer pairs with an efficiency of 90% − 110% were used. The 2^−ΔΔCT^ method [27] was used to calculate gene expression levels, with *gyrB* as the internal standard.

### Construction of mutant and complementation strains

Gene deletion was generated via homologous recombination using the pDM4 plasmid [28]. The target gene upstream and downstream regions (700–850 bp) were amplified. Overlap extension PCR was implemented to fuse the two DNA fragments together. After performing a restriction digest with the appropriate enzymes, the DNA fragment was ligated into pDM4. The resulting plasmid was transformed into *E. coli* S17–1 λ*pir* and next conjugated into RIMD2210633. LB agar containing carbenicillin and chloramphenicol was used for selection of single crossover recombination strain. Counterselection using LB agar containing 15% sucrose was performed to obtain double crossover recombination mutants, which were then verified by PCR and DNA sequencing. The double and triple mutants were constructed using a similar procedure, except that the recombinant plasmid was transferred into a mutant or a double mutant.

Thereafter, pMMB207, a wide-host-range low-copy-number vector [29] was used to construct the complementation strain. A DNA fragment composed of a target gene and an additional ribosome-binding site was amplified from the RIMD2210633 genome. After enzymes digestion, the DNA fragment was ligated into pMMB207. The resulting plasmid was transformed into *E. coli* S17–1 λ*pir* and next conjugated into Δ*1470*. The complementation strain was selected on agar plates containing carbenicillin and chloramphenicol and verified by PCR analysis.

### Growth under zn-deficient conditions

The *V. parahaemolyticus* strains were grown to an OD_600_ of   2 and diluted 1:500 in LB pretreated with DMSO (i.e. normal conditions), varying concentrations of TPEN (30 μM, 35 μM, or 40 μM), or 35 μM TPEN along with 20 μM Zn, respectively. The cultures were transferred into 96-well plates containing 200 μL per well and three wells for each culture. The cultures were incubated at 37°C and 120 rpm. The OD_595_ values were measured hourly with a microplate reader.

### Bacterial motility assay

The swimming and swarming motility assays were performed as previously described with some modifications [30,31]. To analyse the swimming motility, the colonies of each strain were pierced into the semi-solid agar plates (i.e. LB containing 0.3% agar) supplemented with DMSO, 35 μM TPEN, or 35 μM TPEN along with 20 μM Zn, respectively. The plates were photographically documented after incubating at 37°C for 18 h. To evaluate swarming, the *V. parahaemolyticus* strains were grown to an OD_600_ of 0.7; bacterial suspensions of each strain (1 μL) were spotted onto the agar plates (i.e. brain heart infusion (BHI) containing 1.5% agar, 50 μM 2,2´-Bipyridyl and 4 mM CaCl_2_) supplemented with 35 μM TPEN. The plates were photographically recorded after incubating at 24°C for 16 h.

### Mouse infection experiment

The animal experiments were approved by the Animal Welfare and Ethics Committees of Yangzhou University and performed as previously described [21].

40 female C57BL/6 mice (8–12 weeks old) were randomly divided into four groups (10 mice per group). The WT, Δ*1470*, and Δ*1470*Δ*4160*Δ*21425* were cultured in LB broth for 12 h at 30°C and adjusted to 1 × 10^9^ CFU/mL in PBS, respectively. For each group, the mice were intraperitoneally infected with either PBS or 100 μL of the strains. Mouse survival was recorded twice daily for seven days.

### β-galactosidase activity assays

The promoter of the operon *VP_RS01455* to *VP_RS01475* was cloned into pDM8, a plasmid containing the promoterless *lacZ* gene [32]. The resulting plasmid pDM8-P_1475–1455_ was transformed into *E. coli* S17 λ*pir* and then conjugated into the WT strain and Δ*zur*, respectively. The WT strain and Δ*zur* containing the empty pDM8 plasmid were implemented as the control strains.

β-galactosidase activity assays were performed with some modifications to the previously described method [33,34]. The reporter strains were grown in LB pretreated with DMSO or 35 μM TPEN to an OD_600_ of 0.7. Afterwards, 1 mL of each culture was removed, centrifuged, and resuspended in 1 mL PM buffer (60 mM Na_2_HPO_4_, 40 mM NaH_2_PO_4_, 10 mM KCl, and 1 mM MgSO_4_, 50 mM β-mercaptoethanol, pH 7.0). The bacterial suspensions were measured for the A_600_ values and 200 μL of each suspension was added to 800 μL of PM buffer alongside 100 μL of chloroform and 50 μL of 0.1% SDS. After a vigorous vortex to lyse bacterial cells, the reaction was started by adding 200 μL of o-nitrophenyl-β-galactopyranoside (ONPG, 4 mg/mL in PM buffer). When the reaction mixture turned yellowish, 1 M Na_2_CO_3_ (500 μL) was added to stop the reaction. The supernatant of the mixture was then measured for A_420_. The β-galactosidase activity was calculated using the following formula: A_420_ ×1000 × min^−1^ × ml^−1^ × A_600_^−1^.

### Bioinformatic and statistical analysis

The regulon of Zur was predicted by RegPrecise [35]. Statistical analyses were performed using GraphPad Prism 5 (San Diego, USA). qRT-PCR data and β-galactosidase activity assays were analysed by one-way analysis of variance (ANOVA) with a Bonferroni’s post-test. Mouse survival was analysed by the log-rank test.

## Results

### Regulon of zur is partly conserved in vibrionaceae

**Figure 1.**
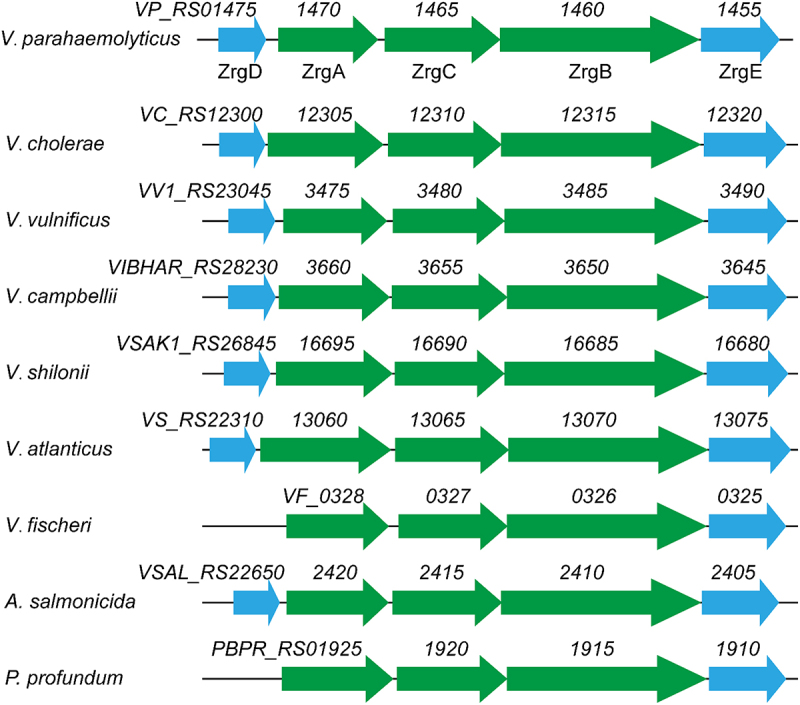
The genetic structures of ZrgABCDE-like systems in *Vibrionaceae*. The structures were based on the following genomes: *V. parahaemolyticus* RIMD2210633, NC_004603.1; *V. cholerae* N16961, NC_002505.1; *V. vulnificus* CMCP6, NC_004459.3; *V. campbellii* ATCC BAA-1116, NC_009783.1; *V. shilonii* AK1 1,103,207,001,947, NZ_ABCH01000030.1; *V. atlanticus* LGP32, NC_011753.2; *V. fischeri* ES114, CP000020.2; *A. salmonicida* LFI1238, NC_011312.1; and *P. profundum* SS9, NC_006370.1.

Bacteria in *Streptococcaceae* use a Zn-binding repressor termed AdcR/ZitR to modulate Zn acquisition, while other bacteria utilize Zur, a regulator belonging to the Ferric uptake regulator (Fur) superfamily [36]. RegPrecise, a resource for genome-scale exploration of transcription factor regulons [35], predicted that the Zur regulon is partly conserved in *Vibrionaceae* (Fig. S1). In *Vibrio cholerae*, dual Zn transporter systems (i.e. ZnuABC and ZrgABCDE) that are regulated by Zur have been characterized [23]. The ZnuABC system, composed of ZnuA – a periplasmic Zn-binding protein, ZnuB – a transmembrane permease, and ZnuC – a cytoplasmic ATPase, is a widespread high-affinity Zn uptake transporter [37,38]. While ZnuABC has been described in many bacterial species, ZrgABCDE has been rarely reported [23,39–43]. Among the *V. cholerae* ZrgABCDE system, ZrgABC is a putative ABC transporter similar to ZnuABC, while ZrgD and ZrgE represent two proteins with unknown functions [23]. In *V. parahaemolyticus*, the loci *VP_RS01455* to *VP_RS01475* form an operon as confirmed by reverse transcription-PCR analysis (Fig. S2). This operon encodes a homolog of the *V. cholerae* ZrgABCDE system. BlastP analysis revealed that ZrgA, ZrgB, ZrgC, ZrgD, and ZrgE in *V. parahaemolyticus* share 49.38%, 82.82%, 61.34%, 40.96%, and 66.67% amino acid identity to their homologs in *V. cholerae*, respectively. The ZrgABCDE-like systems are widely distributed in *Vibrionaceae* (Figure 1). Most species of *Vibrionaceae* encode five proteins in this system, whereas *Vibrio fischeri* and *Photobacterium profundum* lack the ZrgD encoding gene (Figure 1). Multiple sequence alignment of the ZrgA proteins from these species showed that ZrgA possesses several conserved motifs potentially involved in Zn binding (Fig. S3). These results suggest the ZrgABCDE-like system might serve as a common Zn acquisition mechanism in *Vibrionaceae*. In addition to ZnuABC and ZrgABCDE, *V. parahaemolyticus*, *Vibrio vulnificus*, and *Vibrio campbellii* possess a gene cluster that encodes a Zn-regulated TonB-dependent outer membrane receptor, a second ZnuA (designated ZnuA2), and a second ZnuB (designated ZnuB2) (Fig. S1).

### Transcriptional responses of V. parahaemolyticus to Zn limitation

**Figure 2.**
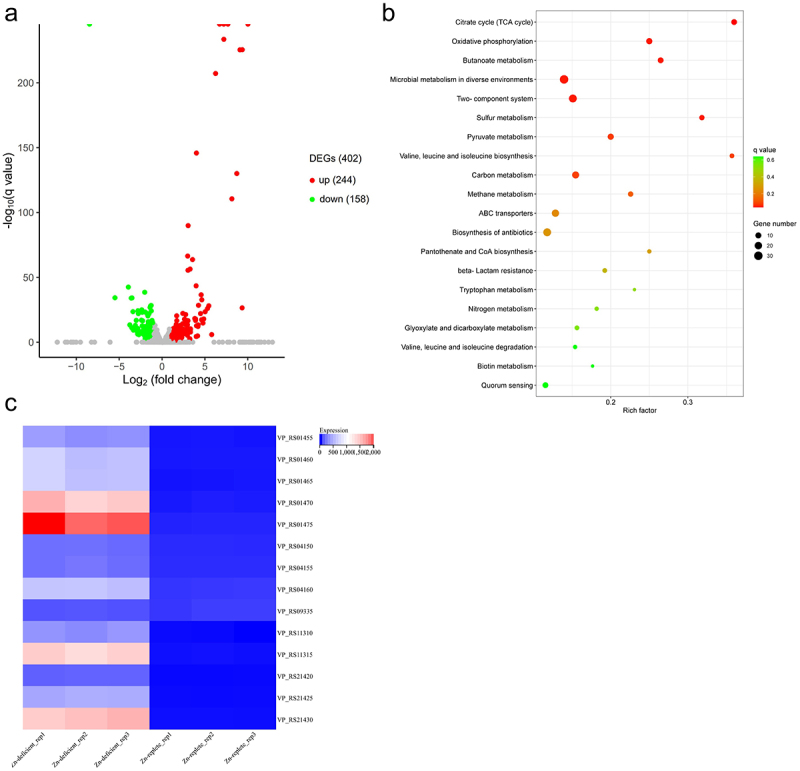
The transcriptome of *V. parahaemolyticus* is altered in response to Zn limitation. (a) the transcriptome change under Zn-deficient conditions compared to Zn-replete conditions. DEGs, up, and down represent the differentially expressed genes, upregulated genes, and downregulated genes, respectively. (b) the top 20 enriched KEGG pathways of the DEGs. The KEGG pathways with a *q* value <0.05 were considered significantly enriched. (c) Heatmap showing the expression of genes in the Zur regulon based on the TPM (transcripts per million) values. Zn-deficient_rep1–3 and Zn-replete 1–3 represent the three biological replications of Zn-deficient and Zn-replete conditions, respectively.

Initially, RNA sequencing was used to reveal the molecular mechanisms of the *V. parahaemolyticus* response to Zn limitation. We compared the transcriptome of *V. parahaemolyticus* RIMD2210633 grown under Zn-deficient (LB pretreated with 35 μM TPEN) and Zn-replete conditions (LB pretreated with 35 μM TPEN along with 500 μM Zn). A total of 402 genes (8.5% of the genome) were differentially expressed during Zn limitation, with 244 genes upregulated and 158 genes downregulated (Figure 2A). The DEGs can be enriched into 70 KEGG pathways, and the top 20 enriched pathways are shown in Figure 2B. Among these, six pathways were significantly enriched, including citrate cycle, oxidative phosphorylation, butanoate metabolism, microbial metabolism in diverse environments, two-component system, and sulphur metabolism (Figure 2B and Table S3). These results indicate that *V. parahaemolyticus* modulates the transcriptome in response to Zn limitation.

### Regulon of V. parahaemolyticus Zur is significantly upregulated under zn limitation conditions

**Figure 3.**
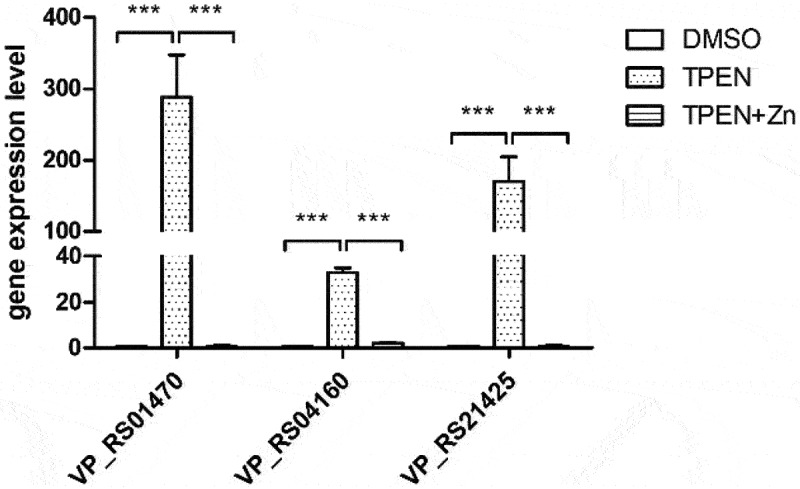
*V. parahaemolyticus* upregulates the expression of genes encoding Zn-binding proteins in response to Zn deficiency. Bar plots representing the mean ± standard deviations of the expression levels of these genes in RIMD2210633 grown in the conditions of interest obtained through quantitative PCR. The data were obtained from three independent experiments. ***, *P* <0.0001.

RNA sequencing analysis revealed that the Zur regulon was significantly upregulated under Zn limitation conditions (Figure 2C and Table 1). The operon *VP_RS21420* to *VP_RS21430*, which encodes a known Zn transporter in *V. parahaemolyticus* [21], was among the most highly upregulated genes (Table 1). The genes *VP_RS04150* to *VP_RS04160*, which encode the conserved Zn transporter ZnuABC, were upregulated approximately 8- to 16-fold (Table 1). Notably, the operon *VP_RS01455* to *VP_RS01475*, which encodes the ZrgABCDE system in *V. parahaemolyticus*, was also highly upregulated (Table 1). qRT-PCR data showed that the expression of *VP_RS01470*, *VP_RS04160*, and *VP_RS21425*, which are annotated to encode Zn-binding proteins, was upregulated approximately 288-, 33-, and 170-fold, respectively, under Zn-deficient conditions, and returned to the baseline levels under Zn-replete conditions (Figure 3). These data are consistent with the RNA sequencing results, and collectively, indicate that the Zur regulon is important for *V. parahaemolyticus* response to Zn limitation.

**Table 1.** Expression of Zur regulon under Zn-deficient conditions compared to Zn-replete conditions.

Gene id	Fold change	Gene name	Gene description
VP_RS01455	75.8352649	*zrgE*	DUF3299 domain-containing protein
VP_RS01460	105.2348993	*zrgB*	ABC transporter permease
VP_RS01465	147.8733108	*zrgC*	ABC transporter ATP-binding protein
VP_RS01470	205.8130005	*zrgA*	DUF2796 domain-containing protein/Predicted zinc-binding protein
VP_RS01475	147.2656093	*zrgD*	DUF2607 domain-containing protein
VP_RS04150	8.2713958	*znuB*	zinc ABC transporter permease subunit ZnuB
VP_RS04155	7.959515461	*znuC*	zinc ABC transporter ATP-binding protein ZnuC
VP_RS04160	16.16829765	*znuA*	zinc ABC transporter substrate-binding protein ZnuA
VP_RS09335	2.1031618	*ribA*	GTP cyclohydrolase II
VP_RS11310	648.4014599	*rpmJ2*	type B 50S ribosomal protein L36
VP_RS11315	656.141252	*rpmE2*	type B 50S ribosomal protein L31
VP_RS21420	283.3969466	*znuBII*	metal ABC transporter permease
VP_RS21425	543.025974	*znuAII*	zinc ABC transporter substrate-binding protein
VP_RS21430	1033.615741	*omr*	Zinc-regulated TonB-dependent outer membrane receptor

### Deletion of VP_RS01470 impairs V. parahaemolyticus growth under zn-deficient conditions

To determine whether *VP_RS01470* involves in Zn acquisition alongside revealing the interactions of the three Zn-binding proteins, we constructed mutants for these Zn-binding proteins in *V. parahaemolyticus* RIMD2210633. These included the single mutants (Δ*1470*, Δ*4160*, and Δ*21425*), the double mutants (Δ*1470*Δ*4160*, Δ*1470*Δ*21425*, and Δ*4160*Δ*21425*), and the triple mutant (Δ*1470*Δ*4160*Δ*21425*). All these mutants were confirmed by PCR (Fig. S4) and DNA sequencing.

**Figure 4.**
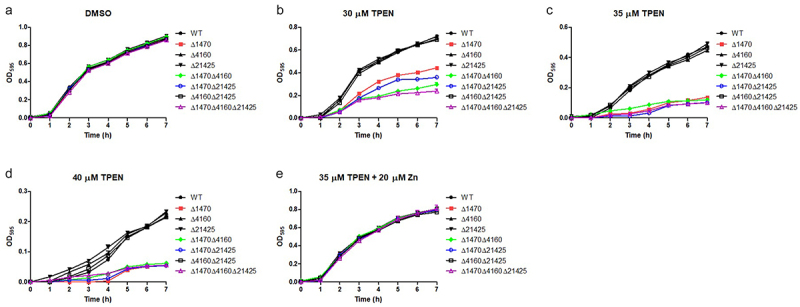
*VP_RS01470* is required for *V. parahaemolyticus* growth under Zn-deficient conditions. (a-d) the strains were grown in LB pretreated with DMSO (a), 30 μM (b), 35 μM (c), or 40 μM TPEN (d). (e) the strains were grown in TPEN (35 μM)-treated LB supplemented with 20 μM Zn. At least three independent experiments were performed for each condition. The data represent the mean ± standard deviations from three wells in an independent experiment.

The growth of RIMD2210633 and its derived mutants were evaluated under Zn-deficient and Zn-replete conditions. All the strains grew well and exhibited almost identical growth curves when cultured in LB pretreated with DMSO (Figure 4A). However, when grown in LB pretreated with TPEN, the mutants lacking either *VP_RS01470* alone or *VP_RS01470* together with other single or multiple genes, exhibited distinctly impaired growth (Figure 4B-D). Notably, when cultured in LB pretreated with 30 μM TPEN, Δ*1470* grew better than the double and triple mutants containing a *VP_RS01470* deletion (Figure 4B). Comparatively, the other three mutants showed similar growths compared to the WT strain (Figure 4B-D). Furthermore, adding 20 μM Zn to TPEN (35 μM)-treated LB restored the growth of the mutants lacking *VP_RS01470* (Figure 4E).

**Figure 5.**
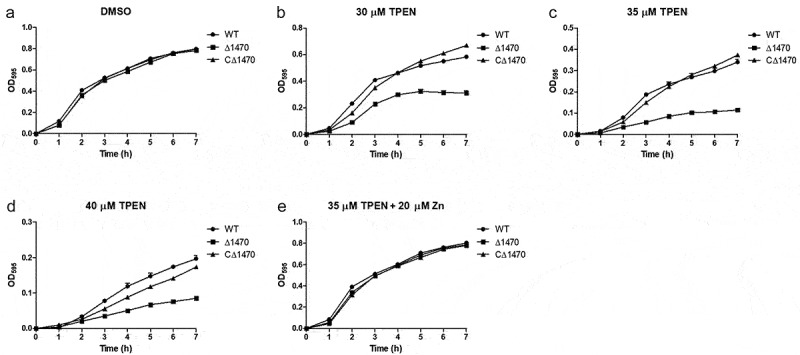
Growth curves of the *V. parahaemolyticus* strains under Zn-deficient and Zn-replete conditions. (a-d) the strains were grown in LB pretreated with DMSO (a), 30 μM (b), 35 μM (c), or 40 μM TPEN (d). (e) the strains were grown in TPEN (35 μM)-treated LB supplemented with 20 μM Zn. At least three independent experiments were performed for each condition. The data represent the mean ± standard deviations from three wells in an independent experiment.

To demonstrate that the growth defect of Δ*1470* is not due to polar effects or undetected mutations outside the gene, we complemented the mutant using plasmid pMMB207-*1470*, and repeated the growth experiments. As seen in Figure 5A-E, CΔ*1470* exhibited growth curves similar to the WT strain under all tested conditions.

Overall, our results indicate that in *V. parahaemolyticus*, *VP_RS01470* plays the predominant role in Zn acquisition, while *VP_RS04160* and *VP_RS21425* play a minor role in Zn acquisition in the case of *VP_RS01470* deletion.

### The reduced swimming motility of Δ1470 under zn-deficient conditions appears to be due to the growth defect of this mutant

**Figure 6.**
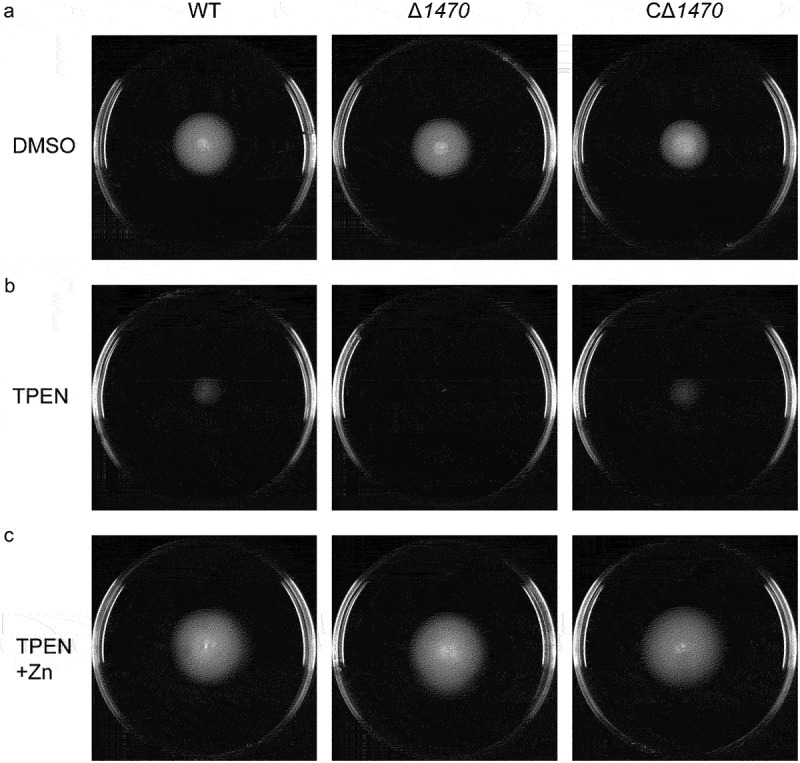
Reduced swimming motility of *VP_RS01470* deletion mutant under Zn-deficient conditions. The strains were grown on LB agar supplemented with DMSO (a), 35 μM TPEN (b), or 35 μM TPEN along with 20 μM Zn (c). The images are representative of at least three independent experiments.

To determine whether *VP_RS01470* involves in the motility of *V. parahaemolyticus*, the WT, Δ*1470*, and CΔ*1470* strains were either pierced into the semi-solid agar or spotted onto the BHI agar supplemented with DMSO, 35 μM TPEN, or 35 μM TPEN along with 20 μM Zn, respectively. The three strains exhibited similar swimming motility on the semi-solid agar plates supplemented with DMSO (Figure 6A). However, when the plates were supplemented with 35 μM TPEN, Δ*1470* exhibited notably reduced swimming motility compared to the WT and CΔ*1470* strains (Figure 6B). Furthermore, the swimming motility of Δ*1470* was restored on the semi-solid agar plates supplemented with 35 μM TPEN along with 20 μM Zn (Figure 6C). Nonetheless, no marked difference in swarming motility was observed between the WT and Δ*1470* strains on the BHI −2,2´-Bipyridyl−CaCl_2_ agar supplemented with 35 μM TPEN (Fig. S5).

**Figure 7.**
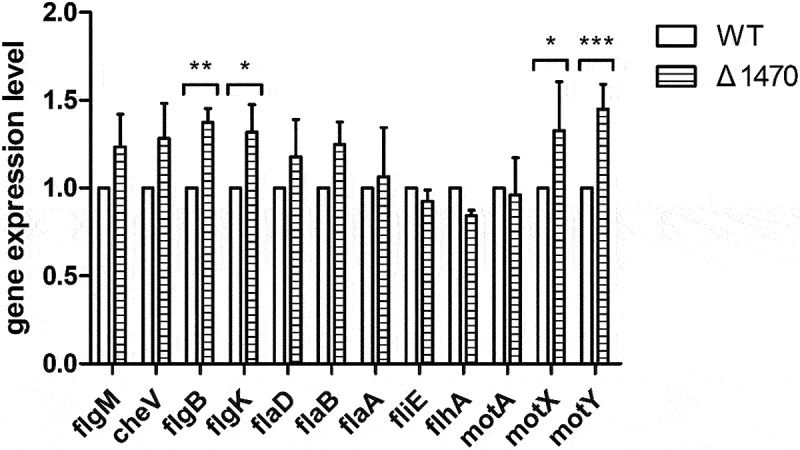
The expression of the polar flagellar-related genes in the WT and Δ*1470* strains grown under Zn-deficient conditions. The first gene in each operon was chosen for quantitative PCR analysis. Bar plots representing the mean ± standard deviations of the expression levels of these genes in the strains of interest. The data were obtained from three independent experiments. *, *P* <0.05; **, *P* <0.01; ***, *P* <0.001.

As the growth of Δ*1470* is severely inhibited under Zn-deficient conditions, the reduced swimming motility of Δ*1470* might be due to the attenuated growth of this mutant. In *V. parahaemolyticus*, the polar flagellum is involved in swimming [44,45]. Therefore, the expression of 12 polar flagellar genes in the WT and Δ*1470* strains grown in LB pretreated with 35 μM TPEN were determined by qRT-PCR analysis. As seen in Figure 7, these genes were not downregulated in Δ*1470* compared to the WT strain.

Taken together, these results suggest that the reduced swimming motility phenotype of Δ*1470* under Zn-deficient conditions seems to be due to the growth defect of this mutant.

### Deletion of VP_RS01470 does not affect V. parahaemolyticus virulence in mice

**Figure 8.**
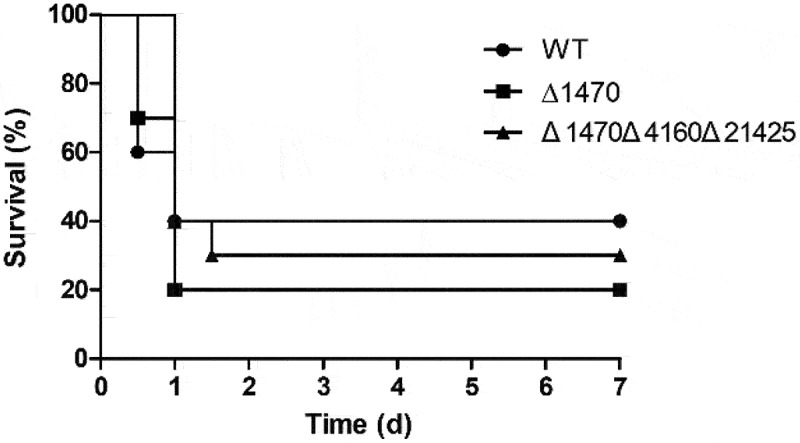
Survival of mice infected with the *V. parahaemolyticus* strains. No significant difference in survival rates was observed between the groups.

A mouse model was employed to evaluate the impact of *VP_RS01470* deletion on *V. parahaemolyticus* virulence. All mice injected with PBS survived over the course of the experiment. As seen in Figure 8, 40% of the WT-infected mice died within 12 h, while 30% of the mice infected with Δ*1470* died within this period. The final survival rates were 40% and 20% for the WT- and Δ*1470*-infected mice, respectively (Figure 8). Furthermore, the mice infected with the triple mutant Δ*1470*Δ*4160*Δ*21425* showed a final survival rate of 30%. There was no significant difference in the survival rates of mice between the different groups. These results indicate that in this model, *VP_RS01470* plays no apparent role in *V. parahaemolyticus* virulence.

### VP_RS01470 is negatively regulated by Zur in V. parahaemolyticus

**Figure 9.**
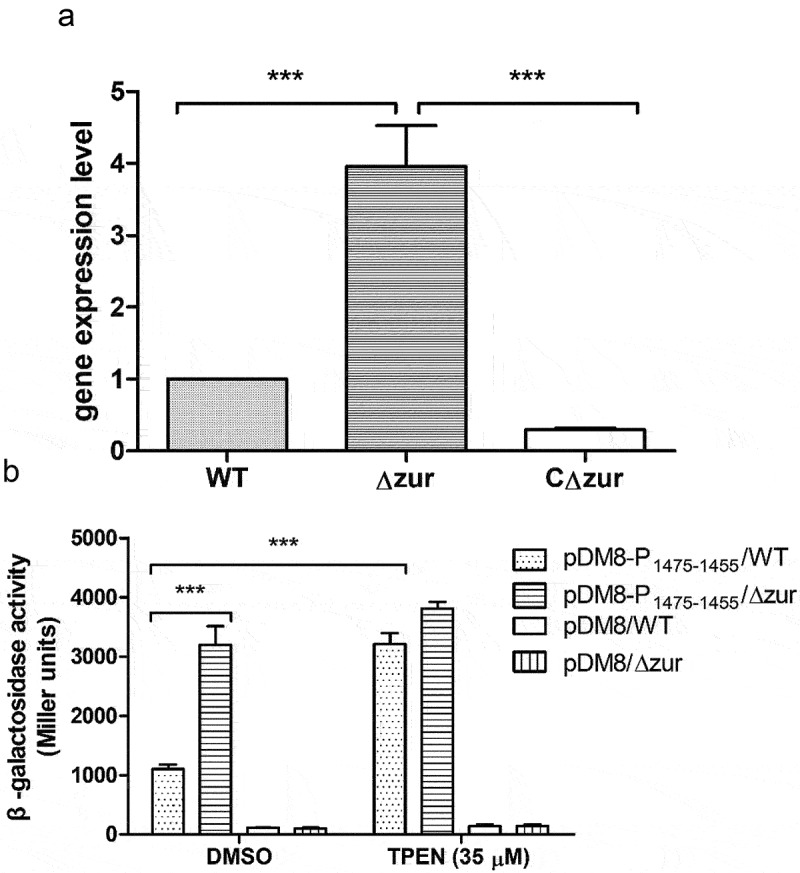
Zur Negatively regulates *VP_RS01470* in *V. parahaemolyticus*. (a) Bar plots representing the mean ± standard deviations of the expression levels of VP_RS01470 in the strains of interest obtained through quantitative PCR. The data were obtained from three independent experiments. (b) β-galactosidase activity of the WT strain or Δ*zur* harbouring either pDM8 or pDM8 containing the promoter of the operon *VP_RS01475* to *VP_RS01455*. The data represent the mean ± standard deviations from three biological replicates.

To confirm the regulation of *VP_RS01470* by Zur, a *zur* deletion mutant (Δ*zur*) and its complementation strain (CΔ*zur*) were constructed (Fig. S4). qRT-PCR data showed that when cultured in LB, *VP_RS01470* expression was significantly upregulated in Δ*zur*, and downregulated in CΔ*zur* (Figure 9A). To further explore the Zur-mediated regulation of *VP_RS01470* in the *V. parahaemolyticus* response to Zn limitation, we constructed LacZ reporter strains and performed β-galactosidase activity assays. When cultured in LB pretreated with DMSO, the β-galactosidase activity of Δ*zur* harbouring the pDM8-P_1475–1455_ plasmid was 2-fold higher than the WT strain harbouring the same plasmid (Figure 9B). Furthermore, the β-galactosidase activity of the WT strain harbouring pDM8-P_1475–1455_ grown in LB pretreated with 35 μM TPEN was approximately 2-fold higher than in LB pretreated with DMSO (Figure 9B). Conversely, the WT strain and Δ*zur* harbouring the empty pDM8 plasmid exhibited only basal levels of β-galactosidase activity, irrespective of the LB treatment (Figure 9B). Altogether, these results reveal that *VP_RS01470* is repressed by Zur in *V. parahaemolyticus* and is derepressed under Zn limitation conditions.

## Discussion

Despite metals playing a crucial role in bacterial physiology and pathogenesis, the mechanisms of metal acquisition in *V. parahaemolyticus* are not fully understood. Here, the transcriptome change of *V. parahaemolyticus* under Zn-deficient conditions was explored in comparison with Zn-replete conditions. Ultimately, a Zn acquisition-associated protein, referred to as ZrgA, was identified in this bacterium. We demonstrated that ZrgA plays a predominant role in Zn acquisition in *V. parahaemolyticus*.

Microbes have evolved complex and diverse mechanisms to respond to metal restriction, including metal acquisition, mobilization, sparing, and recycling [46]. Among these, metal acquisition using high-affinity uptake systems plays a dominant role [13]. Thus, it was unsurprising that the expression of a number of genes was altered under Zn-deficient conditions compared to Zn-replete conditions. Further, the genes encoding Zn transporter systems were among the top upregulated genes. Our results revealed that ZrgA plays the principal role in Zn acquisition in *V. parahaemolyticus*, since only the mutants lacking *VP_RS01470*, rather than *VP_RS04160* and *VP_RS21425*, exhibited a growth defect under the Zn limitation conditions. Moreover, when cultured in LB pretreated with 30 μM TPEN, Δ*1470* grew better than the double mutants lacking *VP_RS01470* and the triple mutant, indicating that during *VP_RS01470* deletion, *VP_RS04160* and *VP_RS21425* can partly complement the function of this gene. Analysis of the WT and various mutants’ growth characteristics demonstrated that *VP_RS01470* plays the predominant role in Zn acquisition in *V. parahaemolyticus*. However, to demonstrate that the growth defect of Δ*1470* is not due to polar effects or undetected mutations outside *VP_RS01470*, we generated a complementation strain for Δ*1470*. Expectedly, the WT and complementation strains exhibited similar growth curves under Zn-deficient conditions.

Gram-positive bacteria generally utilize the AdcABC and AdcAII systems to acquire Zn. These two systems have been demonstrated as essential for Zn acquisition and virulence in *Enterococcus faecalis* and several streptococcal species [11,24,47–52]. In contrast, the contribution of the ZnuABC system to Zn uptake and Zn-dependent physiological processes has been well established in Gram-negative bacteria. For example, ZnuABC is critical for biofilm formation and cell adhesion in enterotoxigenic *E. coli* and contributes to virulence in *Chromobacterium violaceum* [39,41]. *V. cholerae* possesses two Zn uptake systems, namely ZnuABC and ZrgABCDE, where ZnuABC plays the predominant role [23]. Unlike the findings in *V. cholerae*, our results revealed that ZrgA plays the primary role in *V. parahaemolyticus*. Moreover, the ZrgABCDE-like systems are present in various species of *Vibrionaceae*, revealing that *Vibrionaceae* may implement this system as a secondary mechanism for Zn uptake. The product of *VP_RS21425* has been characterized as a Zn transporter acquired by *V. parahaemolyticus* through horizontal gene transfer [21]. However, no growth inhibition was observed when the *VP_RS21425* gene deletion mutant was grown under Zn limitation conditions. Liu et al. used the clinical isolate VP3218 as the WT strain [21], while the RIMD2210633 isolate was used in this study. One hypothesis is that the primary Zn uptake mechanism differs between *V. parahaemolyticus* isolates. In support of this hypothesis, the environmental and virulent *Francisella* species use different Zn acquisition mechanisms to adapt to Zn limitation [53]. Additionally, we could not exclude the possibility that the other two Zn transporters may exert their functions under alternative circumstances.

To adapt to complex and changing environments, *V. parahaemolyticus* has evolved two different types of flagellar systems, i.e. the polar flagellum that is essential for swimming and the lateral flagella that are involved in swarming [44,45]. The regulation of swarming by Ca and Fe has been described previously in *V. parahaemolyticus* [17]. In *Proteus mirabilis*, Zn acquisition contributes to swimming and swarming motility [54]. The Δ*znuABC* mutant of *Salmonella enterica* serovar Typhimurium and *C. violaceum* exhibited decreased swimming motility under Zn limitation conditions [41,55]. In *S. enterica* serovar Typhimurium, a reduction of Zn importation downregulates the expression of flagella, thus leading to reduced motility [55]. Compared to the WT and complementation strains, Δ*1470* exhibited observably reduced swimming rather than swarming motility under Zn limitation conditions. However, the polar flagellar genes were not downregulated in the mutant; thus, the reduced swimming motility observed for Δ*1470* could probably be an artefact of growth defect.

The contribution of Zn acquisition to the pathogenesis has been well established in bacteria [14,21,24,39,41,49,52]. However, ZnuABC is not essential for the virulence of *Y. pestis*, despite that it is the predominant Zn importer during *in vitro* growth [56]. Here, we used a mouse model to examine the impacts of ZrgA deletion on *V. parahaemolyticus* virulence. The results showed that ZrgA plays no noticeable role in the virulence of this bacterium in mice, as the mice infected with either Δ*1470* or the triple mutant exhibited similar survival rates compared to the WT-infected mice. Given that Zn is relatively abundant at various host sites in mice [57], we speculate that *V. parahaemolyticus* might not need the Zn transporters for the process of infection. Alternatively, additional Zn acquisition mechanisms might play a role during the infection of mice. It should be noted that the expression of *VP_RS01470* in *V. parahaemolyticus* isolated from the caecal fluid of infant rabbits is approximately 16-fold higher than from LB cultures [58]. The role of ZrgA in *V. parahaemolyticus* virulence can be investigated in future research using an infant rabbit model.

In *V. cholerae*, Zur negatively regulates the operon *zrgABCDE* in response to limited Zn [23]. Consistent with the observations in *V. cholerae*, the *zrgA* expression was significantly upregulated in Δ*zur* of *V. parahaemolyticus*. Moreover, the β-galactosidase activity suggests that *zrgA* is repressed by Zur under normal conditions and derepressed in Δ*zur* or under Zn-deficient conditions. The mechanism of Zur regulation has been well illustrated in other bacteria, whereby under Zn-replete conditions, Zur binds to DNA and represses the expression of genes encoding Zn transporters. Alternatively, under Zn-deficient conditions, Zur loses its DNA-binding affinity, causing derepression of the target genes [59,60]. Analysis with RegPrecise identified of a putative Zur-binding site―TGAGTGTTATATTATAACACCCG in the upstream of the *zrgABCDE* operon. Consequently, it is likely that Zur regulates the *zrgABCDE* operon via a similar mechanism in *V. parahaemolyticus*.

In conclusion, *V. parahaemolyticus* upregulates the expression of Zur regulon in response to Zn limitation. Moreover, this bacterium prefers ZrgA to uptake Zn under Zn-deficient conditions.

## Supplementary Material

Supplemental Material

## Data Availability

The authors confirm that the data supporting the findings of this study are available within the article and its supplementary materials
