# Supplementary material for: ZrgA contributes to zinc acquisition in Vibrio parahaemolyticus
Source: Virulence. 2023 Jan 4;14(1):2156196. doi: 10.1080/21505594.2022.2156196 (PMC9817125; doi:10.1080/21505594.2022.2156196)
Supplement: Supplemental Material [file KVIR_A_2156196_SM3968.docx]

**Supplementary Table S1.** Bacterial strains and plasmids used in this study.

| Strain or plasmid | Relevant characteristics^a^ | Source or reference |
| --- | --- | --- |
| Strains |  |  |
| *V. parahaemolyticus* |  |  |
| RIMD2210633 | Clinical isolate, Carb^R^ | Makino et al., 2003 |
| Δ*1470* | *VP_RS01470* deletion mutant of RIMD2210633 | This study |
| Δ*4160* | *VP_RS04160* deletion mutant of RIMD2210633 | This study |
| Δ*21425* | *VP_RS21425* deletion mutant of RIMD2210633 | This study |
| Δ*1470*Δ*4160* | *VP_RS01470* and *VP_RS04160* double mutant of RIMD2210633 | This study |
| Δ*1470*Δ*21425* | *VP_RS01470* and *VP_RS21425* double mutant of RIMD2210633 | This study |
| Δ*4160*Δ*21425* | *VP_RS04160* and *VP_RS21425* double mutant of RIMD2210633 | This study |
| Δ*1470*Δ*4160*Δ*21425* | *VP_RS01470*, *VP_RS04160* and *VP_RS21425* triple mutant of RIMD2210633 | This study |
| CΔ*1470* | Complementation strain of Δ*1470* | This study |
| Δ*zur* | *zur* deletion mutant of RIMD2210633 | This study |
| CΔ*zur* | Complementation strain of Δ*zur* | This study |
| pDM8-P_1475-1455_/WT | RIMD2210633 harboring pDM8-P_1475-1455_ | This study |
| pDM8-P_1475-1455_/Δ*zur* | Δ*zur* harboring pDM8-P_1475-1455_ | This study |
| pDM8/WT | RIMD2210633 harboring pDM8 | This study |
| pDM8/Δ*zur* | Δ*zur* harboring pDM8 | This study |
| *E. coli* |  |  |
| DH5α λpir | Cloning host for recombinant vector | Laboratory collection |
| S17-1 λpir | Conjugal donor for recombinant vector | Laboratory collection |
| Plasmids |  |  |
| pDM4 | Suicide vector containing a *sacB* counterselectable marker; Cm^R^ | Milton et al., 1996 |
| pDM4-Δ*1470* | Knockout vector for *VP_RS01470* deletion | This study |
| pDM4-Δ*4160* | Knockout vector for *VP_RS04160* deletion | This study |
| pDM4-Δ*21425* | Knockout vector for *VP_RS21425* deletion | This study |
| pDM4-Δ*zur* | Knockout vector for *zur* deletion | This study |
| pMMB207 | Wide-host-range low-copy-number vector; Cm^R^ | Morales et al., 1991 |
| pMMB207-*1470* | pMMB207 containing *VP_RS01470* and an additional ribosome-binding site | This study |
| pMMB207-*zur* | pMMB207 containing *zur* and an additional ribosome-binding site | This study |
| pDM8 | Plasmid containing the promoterless *lacZ* gene; Cm^R^ | Croxatto et al., 2002 |
| pDM8-P_1475-1455_ | pDM8 containing the promoter of the operon *VP_RS01455* to *VP_RS01475* | This study |

**Supplementary Table S2.** Primers used in this study.

| Primer | Sequence (5'-3')^a^ | Size (bp) | Target gene |
| --- | --- | --- | --- |
| 1455-F | CAAGAGGAATCACGAAGCC | 3168 | An internal region of the operon *VP_RS01455* to *VP_RS01475* |
| 1475-R | AATCCCTGTGCCGTCATAC |  |  |
| Q1470-F | GCATTTTCTGGTGCGTGTTCA | 143 | An internal region of *VP_RS01470* |
| Q1470-R | ACAGCAGAAGAATACCGCCAAC |  |  |
| Q4160-F | TCGCATGATCACGACGGTC | 207 | An internal region of *VP_RS04160* |
| Q4160-R | TGGTGCTAACTGCTGCTCTATTTC |  |  |
| Q21425-F | CCTGTAGCATAGGCAACCACCC | 115 | An internal region of *VP_RS21425* |
| Q21425-R | CAAGATCCACATTACGTGCAAGC |  |  |
| QgyrB-F | TGACAGCCGTTGTTTCGGTA | 273 | An internal region of *gyrB* |
| QgyrB-R | AGTCTGCAAGTTTGCCTGGT |  |  |
| 1470-LA-F | TCC**CCCGGG**ACGCAGGATGCCCTATTAG | 748 | The left arm of *VP_RS01470* |
| 1470-LA-R | TACCGCCAACTTCATTGTGCCCAAGTCG |  |  |
| 1470-RA-F | CACAATGAAGTTGGCGGTATTCTTCTGC | 723 | The right arm of *VP_RS01470* |
| 1470-RA-R | CCG**CTCGAG**GCATTGACGCAAGACGAG |  |  |
| 1470-in-F | TCATGTTTGTCGTGATCGTG | 296 | An internal region of *VP_RS01470* |
| 1470-in-R | TTTAATATTGCCCAAGACGG |  |  |
| 1470-out-F | GTCATAGTAAATCACCAAAGCG | 616/1012 | A fragment containing *VP_RS01470* |
| 1470-out-R | CACCAGAGCATCATACCCAT |  |  |
| 4160-LA-F | C**GAGCTC**CTGAGTGATCGTTGCTGGTG | 772 | The left arm of *VP_RS04160* |
| 4160-LA-R | TGTTGCCTAACGTACTGTTATCGCCTGCGC |  |  |
| 4160-RA-F | GATAACAGTACGTTAGGCAACATGGCTGACA | 782 | The right arm of *VP_RS04160* |
| 4160-RA-R | CCG**CTCGAG**TTTCGACCAGAATCGCATC |  |  |
| 4160-in-F | GCTGCGACCTTCTGATGTAA | 469 | An internal region of *VP_RS04160* |
| 4160-in-R | ATCGGGAGTCACCGTAAAGT |  |  |
| 4160-out-F | CTCCGTTGGGACCGATTA | 545/1311 | A fragment containing *VP_RS04160* |
| 4160-out-R | ATCAGGACAAGTTGGCGAA |  |  |
| 21425-LA-F | C**GAGCTC**GAAGCTGCTATCCCCAACA | 856 | The left arm of *VP_RS21425* |
| 21425-LA-R | GCAAATATCTATCTCACCAAGATCAACGCC |  |  |
| 21425-RA-F | TCTTGGTGAGATAGATATTTGCTTCTGGAGCG | 791 | The right arm of *VP_RS21425* |
| 21425-RA-R | CCG**CTCGAG**AGATGTCGGCAGTGGATGA |  |  |
| 21425-in-F | CGAACTTGCGTCCAACTTT | 510 | An internal region of *VP_RS21425* |
| 21425-in-R | GCCGATTTGGCTATGTGTT |  |  |
| 21425-out-F | TAACGACATTATCCAAGGGC | 691/1295 | A fragment containing *VP_RS21425* |
| 21425-out-R | CTTCGCACTTTGGTACTGTTC |  |  |
| QflgM-F | GCCGCTACTTTCGCACTGTC | 112 | An internal region of *VP_RS03800* |
| QflgM-R | AAGCGCCAGCTCAACAAGAC |  |  |
| QcheV-F | GACTCAACGGTTGCACGTAAGC | 123 | An internal region of *VP_RS03810* |
| QcheV-R | GCTGCCTTCTTGAGCCATTTC |  |  |
| QflgB-F | CCAAGGAGTTACAAGCGGCA | 131 | An internal region of *VP_RS03820* |
| QflgB-R | ACCCGTGTCTGGTTGCGTAG |  |  |
| QflgK-F | GCACTCCTGGTCCACATAACGAT | 141 | An internal region of *VP_RS03865* |
| QflgK-R | CCAAGGTATGACCATTGCCGAT |  |  |
| QflaD-F | ATTCTCTGGCGGTCTGTCTGG | 149 | An internal region of *VP_RS03880* |
| QflaD-R | CACGGTGGCTGTCTACGTATTTC |  |  |
| QflaB-F | TTGATACGGCTCTTCGACGC | 119 | An internal region of *VP_RS10970* |
| QflaB-R | CTGAAATACGTAGACAGCCACCG |  |  |
| QflaA-F | GGTTGCTGATTGCGTGGTTG | 117 | An internal region of *VP_RS10965* |
| QflaA-R | CGGTTGCAGGCTCTCAAGAAG |  |  |
| QfliE-F | CTCAACGAGCTTATTGCGGATT | 101 | An internal region of *VP_RS10925* |
| QfliE-R | GTGGTGATGCCGATGTTTCC |  |  |
| QflhA-F | GATGCGTTGCCAATACCGTC | 131 | An internal region of *VP_RS10850* |
| QflhA-R | GATTGATGGCGAACCGACC |  |  |
| QmotA-F | GGTGGTTTTGCCTTCGTCATC | 138 | An internal region of *VP_RS03305* |
| QmotA-R | AAAGAACTGGCCGAGCGTG |  |  |
| QmotX-F | TAACCCACGCCTTAACACGG | 119 | An internal region of *VP_RS13775* |
| QmotX-R | TGCTGCTACGCGATTACGGT |  |  |
| QmotY-F | GTCTTTACCAATCGGCGTGC | 144 | An internal region of *VP_RS10270* |
| QmotY-R | GAAAGCCAGAGCCTATCGGAG |  |  |
| zur-LA-F | C**GAGCTC**CGTCAATGCCAATACGTGTT | 717 | The left arm of *zur* |
| zur-LA-R | ACATCACCACATTTCACCATAACTATCGGACG |  |  |
| zur-RA-F | TTATGGTGAAATGTGGTGATGTCGTTGAACTC | 830 | The right arm of *zur* |
| zur-RA-R | CCG**CTCGAG**GGCTAACCAAATCCTACCTGA |  |  |
| zur-in-F | CGCAGAGCGTGGAGTAAG | 248 | An internal region of *zur* |
| zur-in-R | ATTGGAAAAAGTGCTGTTGAG |  |  |
| zur-out-F | CGTTCTGTGATGGCTATTCC | 442/748 | A fragment containing *zur* |
| zur-out-R | AACCAGAAACGTCACCTCG |  |  |
| C1470-F | CCG**GAATTC**TAAGGAGGTAGGATAATAATGCCATCTAAACAGGTTTTAGC | 630 | *VP_RS01470* and an additional ribosome-binding site |
| C1470-R | ACGC**GTCGAC**TTATTTGATTGCGATTTGAGTG |  |  |
| Czur-F | CGG**GGTACC**TAAGGAGGTAGGATAATAATGGTGAAAGGTTTGAACCC | 465 | *zur* and an additional ribosome-binding site |
| Czur-R | ACGC**GTCGAC**CTATACTTTTTCTTTCGTCTCGG |  |  |
| P_1475-1455_-F | cggatccggggaattcccgggCTCCTGACTCGGCACCAATC | 145 | The promoter of the operon *VP_RS01455* to *VP_RS01475* |
| P_1475-1455_-R | aagcttatcgattcgcccgggTGAACGCCTGAAATCCTAACG |  |  |

^a^ The bold sequences are restriction sites.

**Supplementary Table S3.** The significantly enriched KEGG pathways.

| Term | ID | Input number | Background number | *q* value |
| --- | --- | --- | --- | --- |
| Citrate cycle (TCA cycle) | vpa00020 | 9 | 25 | 0.040830848 |
| Oxidative phosphorylation | vpa00190 | 11 | 44 | 0.049120052 |
| Butanoate metabolism | vpa00650 | 9 | 34 | 0.049120052 |
| Microbial metabolism in diverse environments | vpa01120 | 30 | 215 | 0.049120052 |
| Two-component system | vpa02020 | 24 | 159 | 0.049120052 |
| Sulfur metabolism | vpa00920 | 7 | 22 | 0.049120052 |


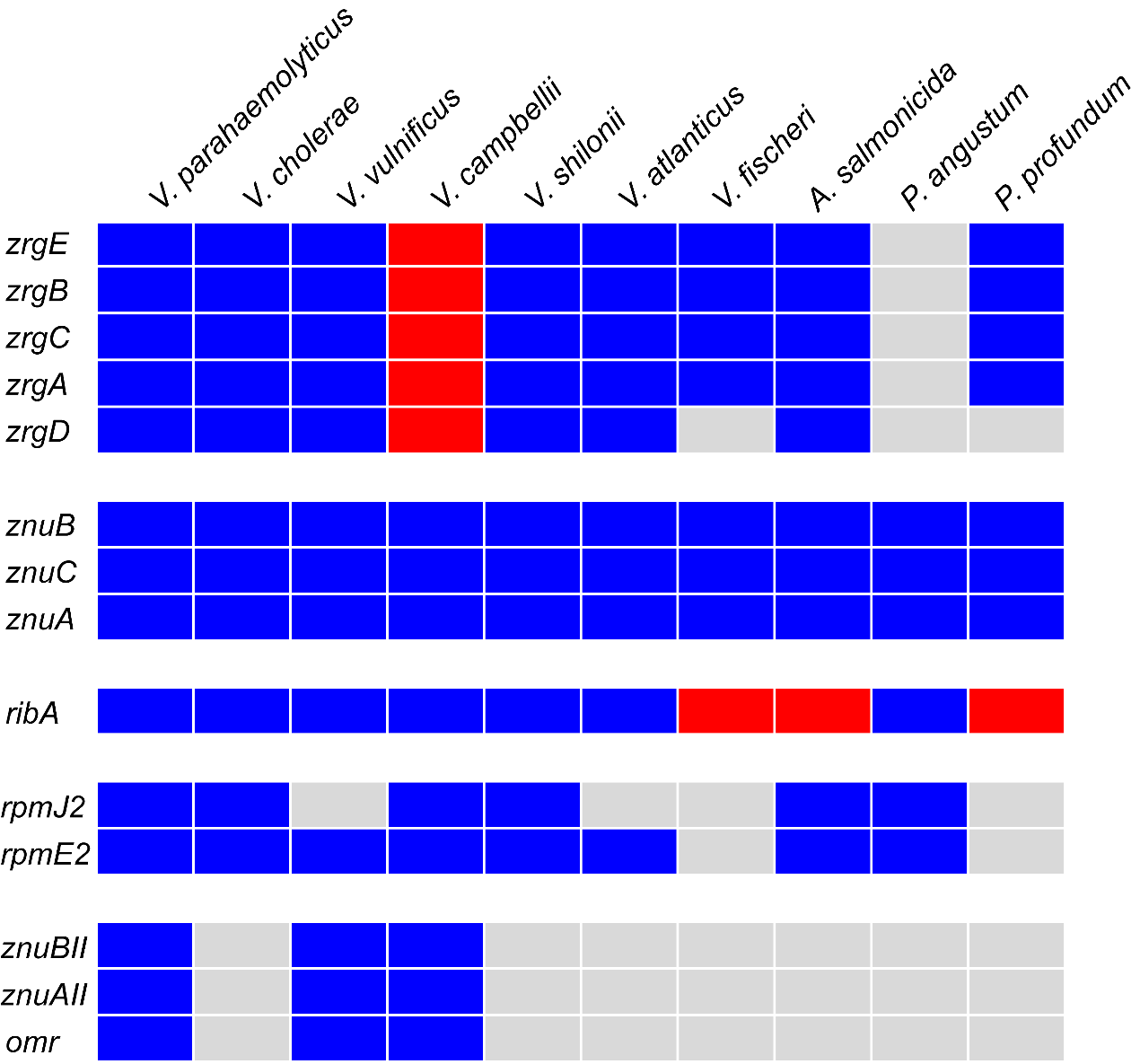


**Supplementary Figure 1.** The regulon of Zur in *Vibrionaceae*. Blue colour, the gene is in the regulon; red colour, the gene is not in the regulon; grey colour, the homologous gene is absent. The analysis was based on the following genomes: *V. parahaemolyticus* RIMD2210633, NC_004603.1; *V. cholerae* N16961, NC_002505.1; *V. vulnificus* CMCP6, NC_004459.3; *V. campbellii* ATCC BAA-1116, NC_009783.1; *V. shilonii* AK1 1103207001947, NZ_ABCH01000030.1; *V. atlanticus* LGP32, NC_011753.2; *V. fischeri* ES114, CP000020.2; *A. salmonicida* LFI1238, NC_011312.1; *P. angustum* S14, GCA_000153265.1; and *P. profundum* SS9, NC_006370.1.


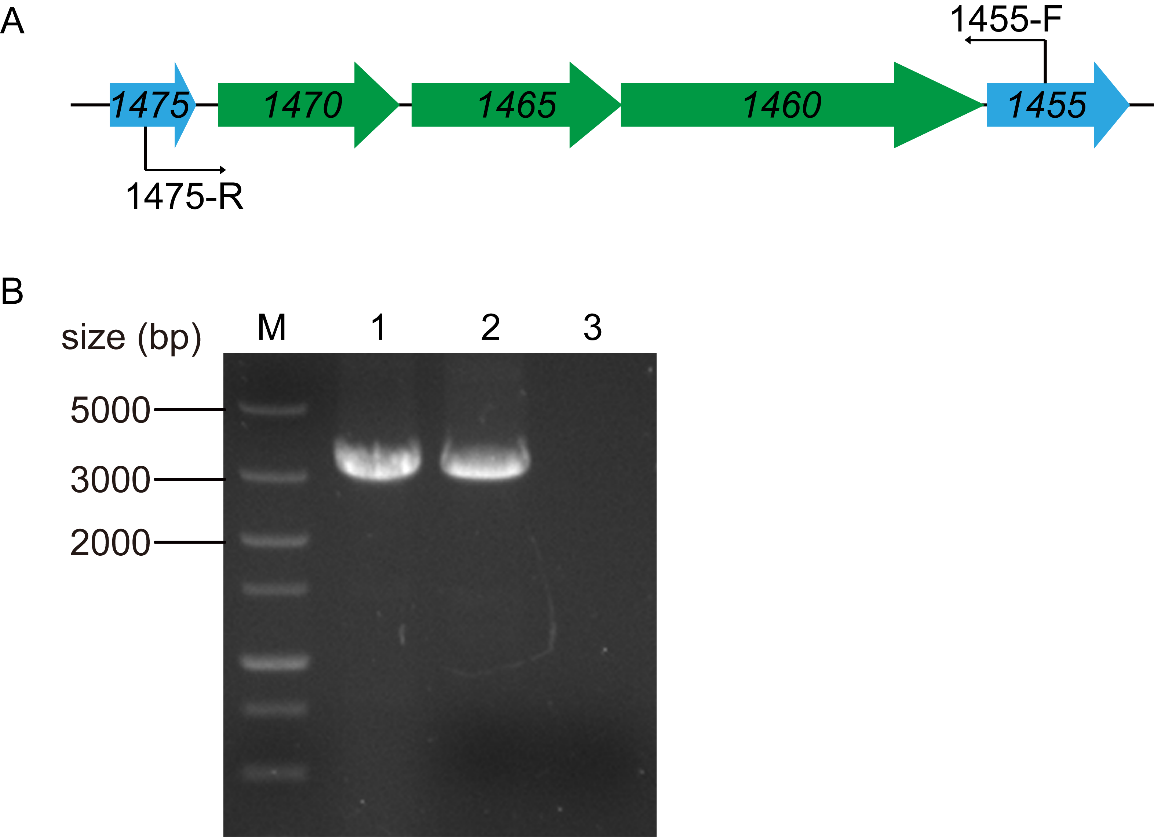


**Supplementary Figure 2.** The loci *VP_RS01455* to *VP_RS01475* form an operon in *V. parahaemolyticus*. The locations of the primers used for reverse transcription-PCR analysis are indicated in (A). (B) PCR was carried out using using genomic DNA (lane 1), cDNA generated from total RNA of RIMD2210633 (lane 2), and cDNA– (cDNA reaction without reverse transcriptase). Lane M indicates the DL 5000 DNA Marker.


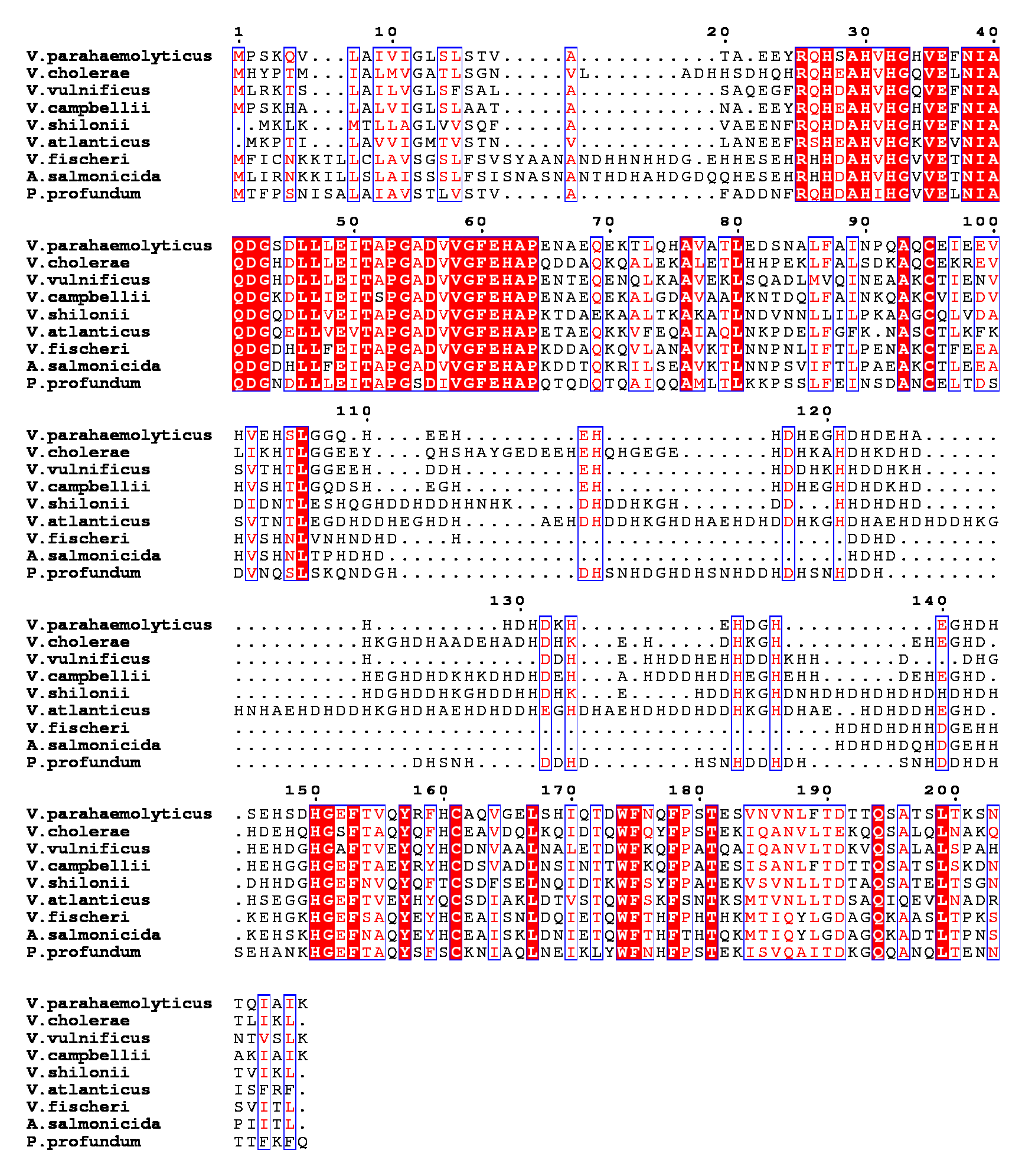


**Supplementary Figure 3.** Multiple sequence alignment of the ZrgA proteins from certain species of *Vibrionaceae*. GenBank accession numbers for the ZrgA proteins are the following: *V. parahaemolyticus*, WP_015296183.1; *V. cholerae*, WP_000559203.1; *V. vulnificus*, WP_011078793.1; *V. campbellii*, WP_012126893.1; *V. shilonii*, WP_006073232.1; *V. atlanticus*, WP_012604885.1; *V. fischeri*, AAW84823.1; *A. salmonicida*, WP_012549253.1; and *P. profundum*, WP_011217165.1.


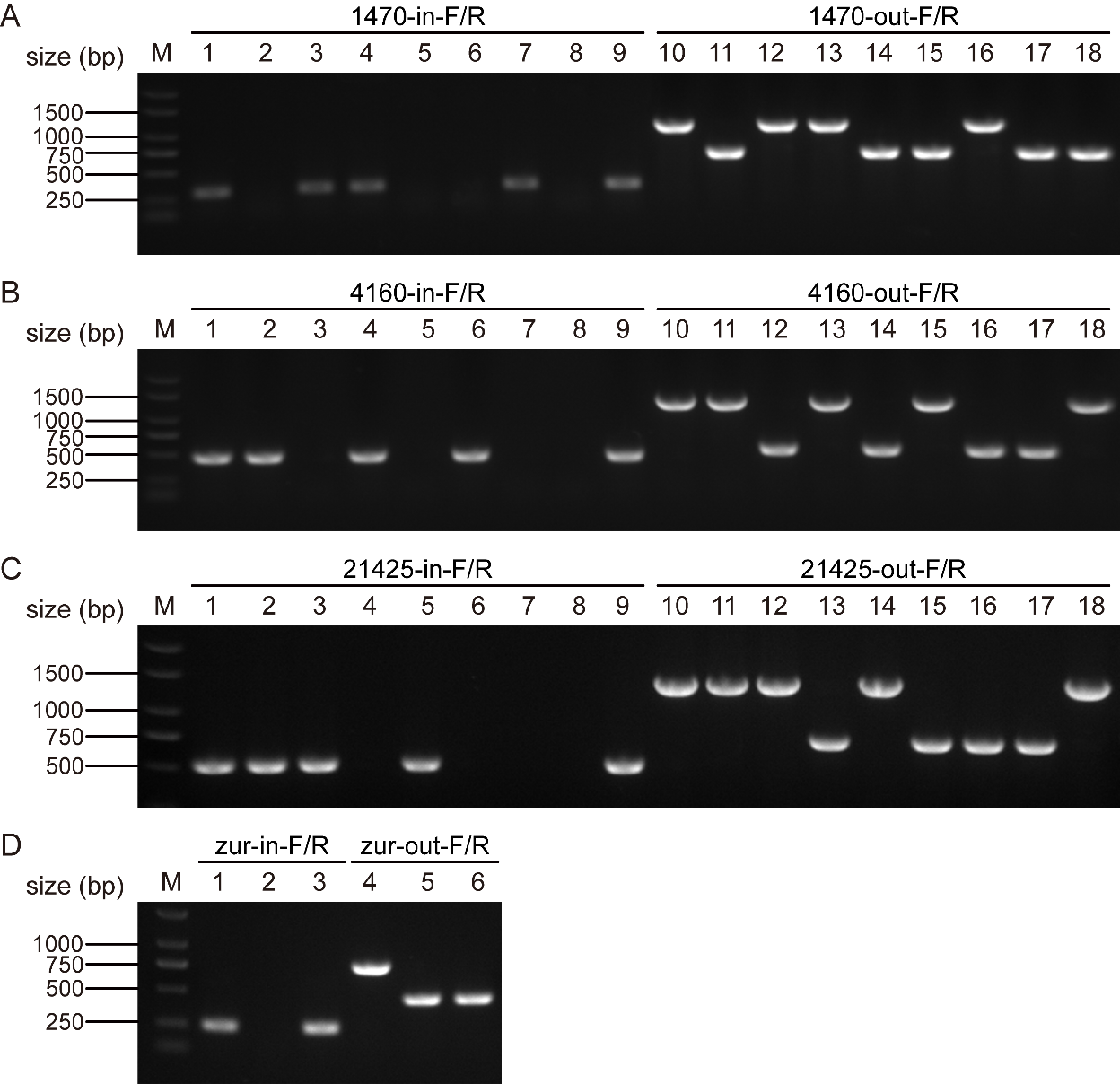


**Supplementary Figure 4.** PCR identification of the mutant and complementation strains. (A-C) PCR amplification of genomic DNAs from the *V. parahaemolyticus* strains using the primer pairs 1470-in-F/R, 1470-out-F/R (A), 4160-in-F/R, 4160-out-F/R (B), and 21425-in-F/R, 21425-out-F/R (C), respectively. Lane M indicates the DL2000 DNA Marker. Lanes 1 and 10, the WT strain; lanes 2 and 11, Δ*1470*; lanes 3 and 12, Δ*4160*; lanes 4 and 13, Δ*21425*; lanes 5 and 14, Δ*1470*Δ*4160*; lanes 6 and 15, Δ*1470*Δ*21425*; lanes 7 and 16, Δ*4160*Δ*21425*; lanes 8 and 17, Δ*1470*Δ*4160*Δ*21425*; lanes 9 and 18, CΔ*1470*. (D) PCR amplification of genomic DNAs from the WT (lanes 1 and 4), Δ*zur* (lanes 2 and 5), and CΔ*zur* strains (lanes 3 and 6) using the primer pairs zur-in-F/R, zur-out-F/R, respectively. Lane M indicates the DL 2000 DNA Marker.


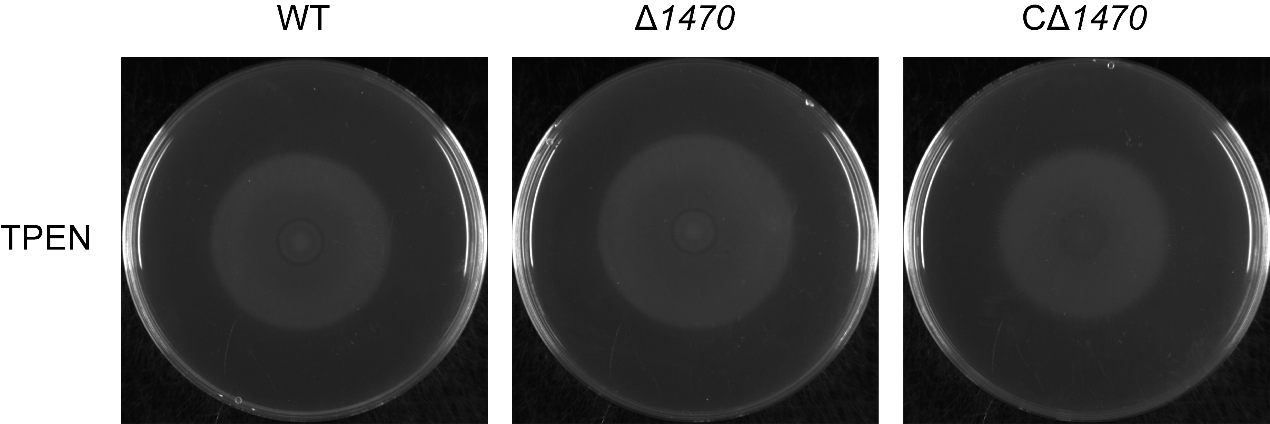


**Supplementary Figure 5.** *VP_RS01470* has no apparent role in the swarming motility of *V. parahaemolyticus*. The strains were grown on the BHI−2,2´-Bipyridyl−CaCl_2_ agar supplemented with 35 μM TPEN. The images are representative of at least three independent experiments.
